# When Hydronephrosis Was Not Physiological: A Case of Septic Shock Secondary to Obstructive Uropathy in Pregnancy

**DOI:** 10.7759/cureus.118251

**Published:** 2026-10-20

**Authors:** Soneya Chowdhury, Wisdom A Amoo, Sumi Surendran, Lisa Pilkington

**Affiliations:** 1 Obstetrics and Gynaecology, Aneurin Bevan University Health Board, Newport, GBR

**Keywords:** maternal sepsis, obstructive hydronephrosis, obstructive urosepsis, pregnancy-related hydronephrosis, urinary stones

## Abstract

Urosepsis in pregnancy is a life-threatening condition requiring early recognition, rapid multidisciplinary escalation and timely source control. Physiological hydronephrosis is common during pregnancy and may obscure clinically significant urinary tract obstruction. We report a 26-year-old gravida 5 para 3 woman at 30 weeks’ gestation who presented with severe right-sided loin pain. Initial ultrasound demonstrated moderate right hydronephrosis without a visible calculus. Within hours, she deteriorated with pyrexia, acute kidney injury, lactic acidosis and septic shock. Computed tomography demonstrated high-grade obstruction of the right renal collecting system secondary to a ureteropelvic junction calculus with associated inflammatory change. Following maternal deterioration and evidence of foetal compromise, an urgent multidisciplinary decision was made to proceed with category 1 caesarean delivery followed by cystoscopy and right ureteric stent insertion. Cultures grew *Klebsiella pneumoniae*. The patient subsequently required intensive care support including invasive ventilation, vasopressors, renal replacement therapy and treatment for disseminated intravascular coagulation. Both maternal and neonatal outcomes were favourable. This case highlights the diagnostic challenge of distinguishing physiological hydronephrosis from pathological obstruction in pregnancy and emphasises the importance of source control in infected obstructive uropathy.

## Introduction

Maternal sepsis remains an important cause of maternal morbidity and mortality worldwide. Urinary tract infection (UTI) is among the most common bacterial infections encountered during pregnancy and represents a spectrum ranging from asymptomatic bacteriuria to severe pyelonephritis. Pyelonephritis may result in sepsis, acute respiratory distress syndrome, disseminated intravascular coagulation (DIC), acute kidney injury and preterm birth [1,2].

Pregnancy predisposes to urinary tract stasis through progesterone-mediated smooth muscle relaxation, ureteric dilatation and mechanical compression by the enlarging uterus, which occurs in 90% of women [3]. Consequently, physiological hydronephrosis occurs commonly, particularly on the right side. Although generally benign, physiological dilatation may create diagnostic uncertainty when pregnant patients present with flank pain because ultrasonography may not reliably distinguish physiological dilatation from significant obstruction [4,5].

Obstructive uropathy complicated by infection is a urological emergency, with an incidence in pregnancy of one in 1500, which is similar to non-pregnant women [3]. In the presence of an obstructed infected collecting system, antimicrobial therapy alone is insufficient and prompt decompression through ureteric stenting or percutaneous nephrostomy is often required to achieve source control. Delays may lead to septic shock, multiorgan dysfunction and adverse maternal-foetal outcomes [6-8].

We present a case of severe septic shock secondary to an obstructed infected kidney during the third trimester of pregnancy, highlighting the importance of repeated reassessment, appropriate imaging, multidisciplinary management and timely source control.

## Case presentation

A 26-year-old woman, gravida 5 para 3, presented to the Maternity Triage Unit at 30 weeks' gestation with acute severe right-sided loin pain radiating to the suprapubic region. The pain was described as colicky in nature and like a previous episode of renal colic during which she had spontaneously passed a renal stone. There were no features suggestive of preterm labour, placental abruption or other obstetric pathology at presentation.

Initial observations demonstrated a heart rate of 93 beats/minute, blood pressure of 104/53 mmHg, temperature of 37.4°C, respiratory rate of 19 breaths/minute and oxygen saturation of 97% on room air. Abdominal examination revealed a soft, non-tender gravid abdomen with no uterine tenderness. No significant renal angle tenderness was elicited. Cardiotocography (CTG) demonstrated a reassuring, non-hypoxic foetal heart rate trace.

Initial investigations showed a white cell count of 12.0 ×10⁹/L, C-reactive protein (CRP) of 11 mg/L, lactate of 1.7 mmol/L, amylase of 34 U/L and normal renal function. Renal tract ultrasonography demonstrated moderate right-sided hydronephrosis without visualisation of an obstructing calculus. The right ureter could not be completely assessed because of interference from overlying bowel gas and the gravid uterus (Figure 1). Although the findings were considered potentially consistent with physiological hydronephrosis of pregnancy, the severity and character of the patient's pain warranted admission for analgesia, observation and further assessment.

**Figure 1:**
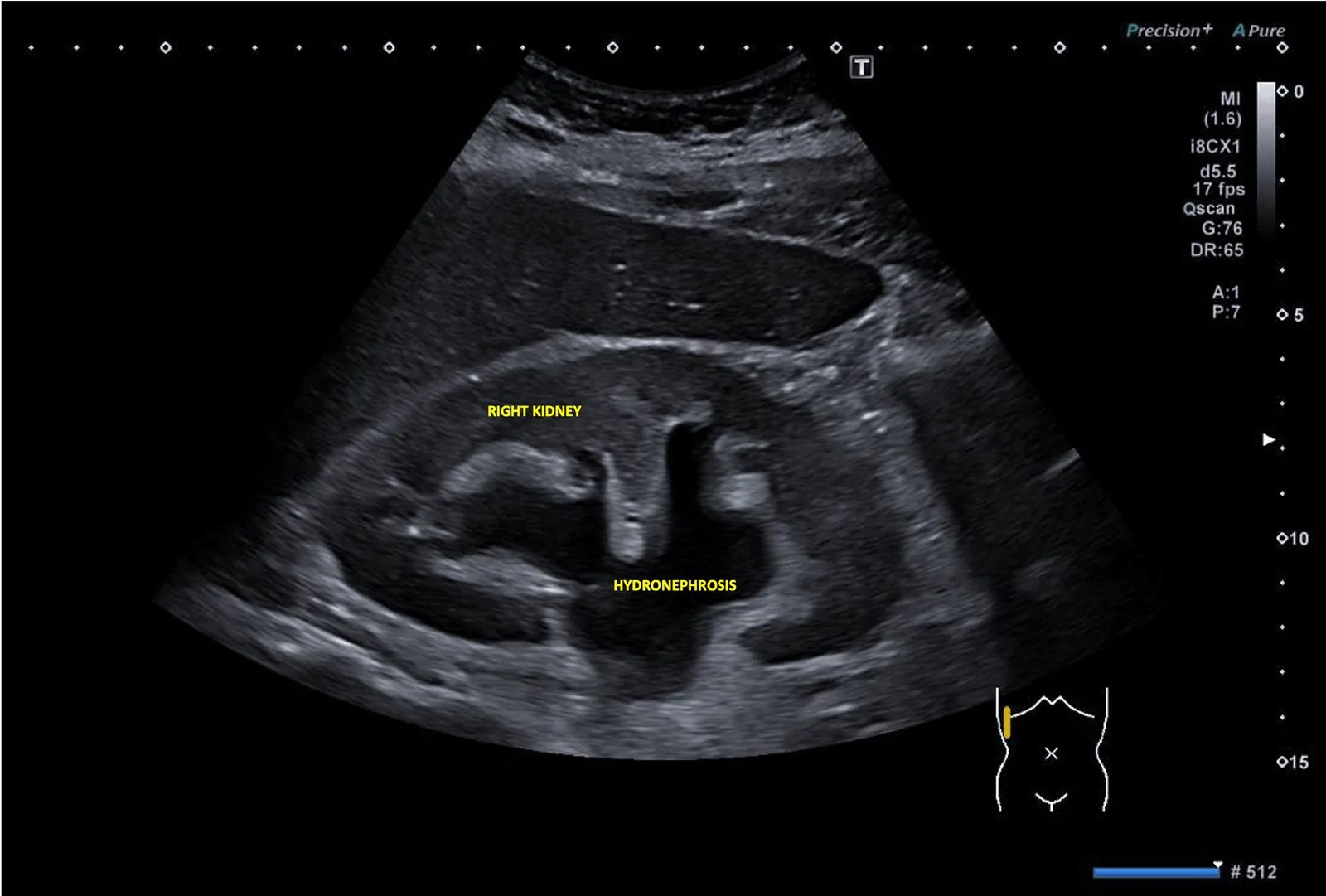
Ultrasound scan image of the right kidney showing right-sided hydronephrosis.

A few hours into admission, the patient deteriorated significantly. She developed worsening loin pain accompanied by nausea, vomiting, pyrexia (40.2°C), sinus tachycardia (140 beats/minute) and tachypnoea (34 breaths/minute). Despite these changes, blood pressure remained relatively preserved at 102/51 mmHg.

Repeat blood investigations demonstrated a rising inflammatory response and evolving organ dysfunction, with a white cell count of 17.8 ×10⁹/L, CRP of 153 mg/L, serum creatinine of 139 μmol/L (baseline 68 μmol/L) and lactate of 3.7 mmol/L. These findings prompted urgent transfer to the maternity high-dependency unit (HDU) for continuous maternal and foetal monitoring.

A maternal sepsis pathway was initiated immediately. Blood cultures were obtained; broad-spectrum intravenous antibiotic therapy with Tazocin (piperacillin-tazobactam) 4.5 g every eight hours was commenced; intravenous fluid resuscitation with Ringer's lactate was initiated; and a urinary catheter was inserted for strict fluid balance monitoring. In view of the rapid clinical deterioration and ongoing concern regarding an underlying surgical pathology, a computed tomography (CT) scan of the abdomen and pelvis was performed. This demonstrated high-grade obstruction of the right renal collecting system caused by a calculus located at the right ureteropelvic junction, with associated perinephric inflammatory change (Figures 2-3). Mild left-sided hydronephrosis and hydroureter were also noted.

**Figure 2:**
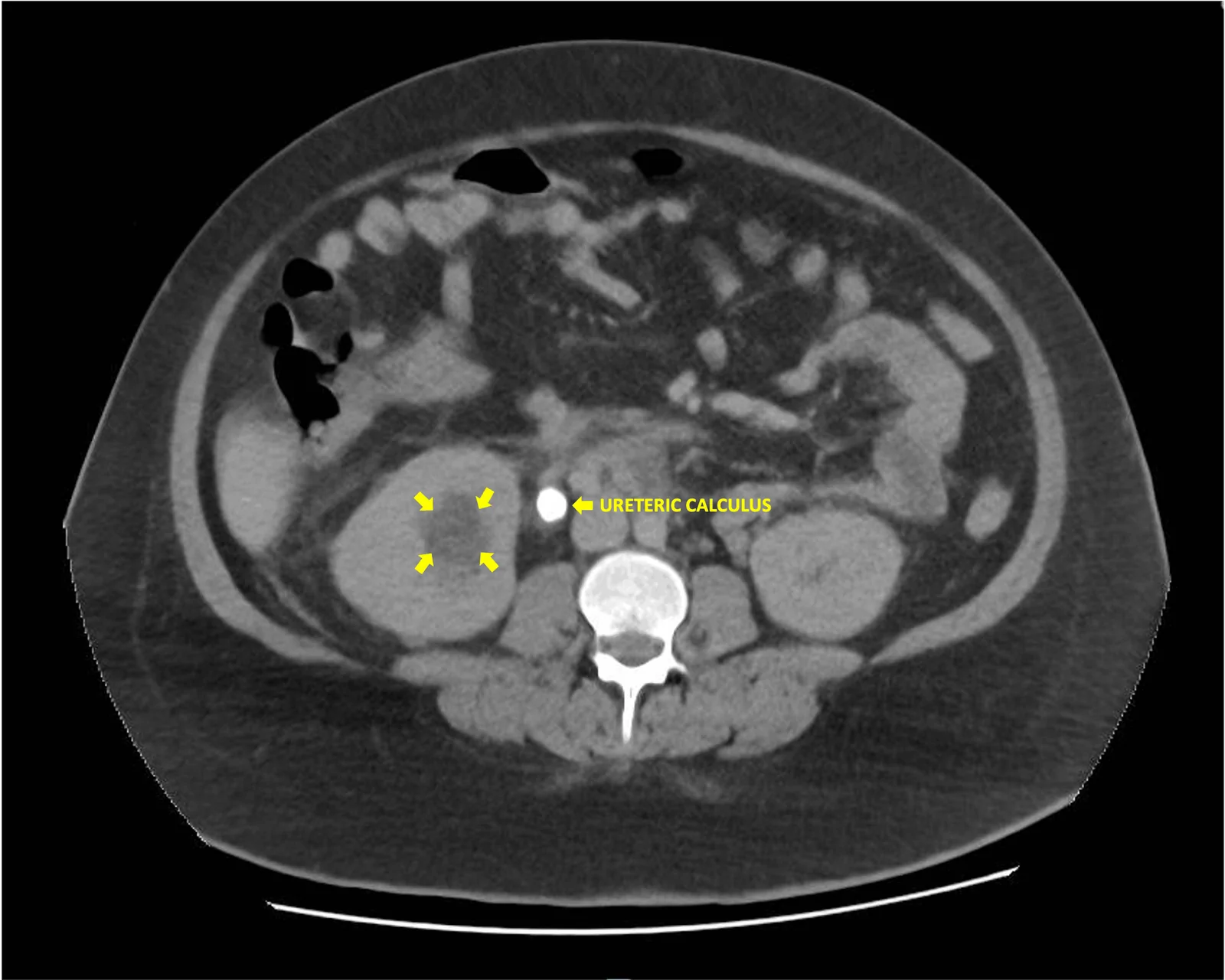
CT scan of the abdomen showing the ureteric calculus at the right ureteropelvic junction, with yellow arrows pointing to the dilated right renal pelvis.

**Figure 3:**
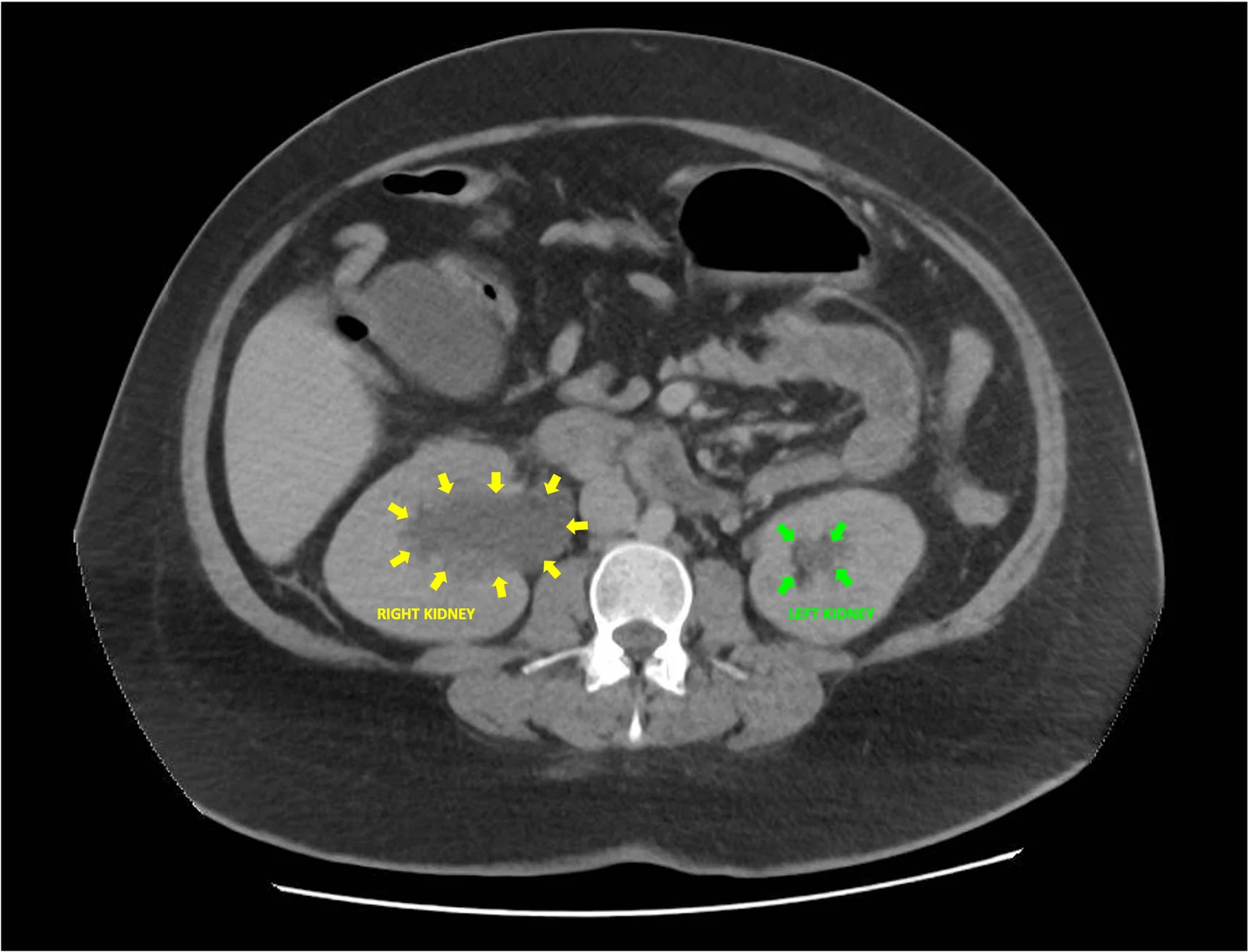
CT scan of the abdomen with yellow arrows pointing to the marked right-sided hydronephrosis due to calculi obstruction, with associated mild left-sided hydronephrosis (green arrows).

Despite resuscitation, the patient progressed to profound septic shock. Blood pressure fell to 92/41 mmHg, with persistent tachycardia of 160-170 beats/minute. Venous blood gas analysis demonstrated severe metabolic acidosis (pH 7.20, bicarbonate 11 mmol/L, lactate 11 mmol/L). Urine output decreased despite fluid resuscitation, and renal function continued to deteriorate. Concurrently, CTG monitoring demonstrated progressive foetal compromise, with the development of a compensated hypoxic trace characterised by a rising baseline foetal heart rate.

Given the combination of maternal septic shock, radiologically confirmed urinary obstruction and worsening foetal condition, an urgent multidisciplinary review was undertaken involving obstetricians, anaesthetists, urologists, intensivists, microbiologists and neonatologists. Following discussion, a decision was made to proceed with emergency category 1 caesarean delivery followed immediately by definitive urological source control.

The patient underwent emergency caesarean section under general anaesthesia and delivered a live preterm infant weighing 1535 g. Apgar scores were 1 at one minute and 4 at five minutes. The neonate was admitted to the neonatal intensive care unit (NICU), where brief ventilatory support was required before successful extubation.

During the same operative episode, the urology team performed cystoscopy and right ureteric stent insertion. Intraoperative findings included a tortuous proximal right ureter at the ureteropelvic junction containing a calculus, with foul-smelling infected urine draining from the right renal collecting system. A 6 Fr × 26 cm ureteric stent was successfully inserted. Samples of aspirated urine were sent for microbiological culture and sensitivity testing, and stone fragments were submitted for compositional analysis (report showed calcium phosphate with traces of calcium oxalate and struvite predominantly).

Postoperatively, pre-surgical clinical status and difficulties with extubation necessitated transfer to the intensive care unit (ICU) for level 3 care. Her ICU course was characterised by multiorgan dysfunction requiring invasive mechanical ventilation, high-dose vasopressor support with noradrenaline and vasopressin, stress-dose corticosteroids, renal replacement therapy and blood product support for DIC.

Empirical treatment with piperacillin-tazobactam was continued initially. Subsequent blood and urine cultures both grew *Klebsiella pneumoniae*, prompting antimicrobial optimisation in conjunction with microbiology advice, and the addition of intravenous gentamicin 240 mg daily.

Her ICU admission was further complicated by right upper limb ischaemia. Doppler ultrasonography demonstrated radial artery thrombosis and occlusion, with preserved ulnar artery perfusion. Following multidisciplinary discussion with vascular surgery and haematology teams, therapeutic intravenous heparin was commenced before later conversion to prophylactic anticoagulation.

Following extubation, the patient developed delirium requiring treatment with quetiapine, which gradually resolved.

Repeat CT imaging demonstrated satisfactory decompression of the right collecting system with appropriate ureteric stent position and no evidence of a perinephric collection (Figure 4). Renal function subsequently improved over the next few days with serum creatinine increasing to a peak of 618 μmol/L before progressively recovering following relief of obstruction and critical care support. Correspondingly, eGFR declined markedly during the acute illness before returning towards baseline during recovery, and the inflammatory markers followed a similar pattern, with the white cell count and CRP peaking at over 40 ×10⁹/L and 350 mg/L, respectively, before gradually normalising. Biochemical markers and trend from admission till discharge outlined in Table 1, trend drawn in Figure 5. Nephrology review concluded that the acute kidney injury was recovering and recommended ongoing monitoring of renal function and fluid balance.

**Figure 4:**
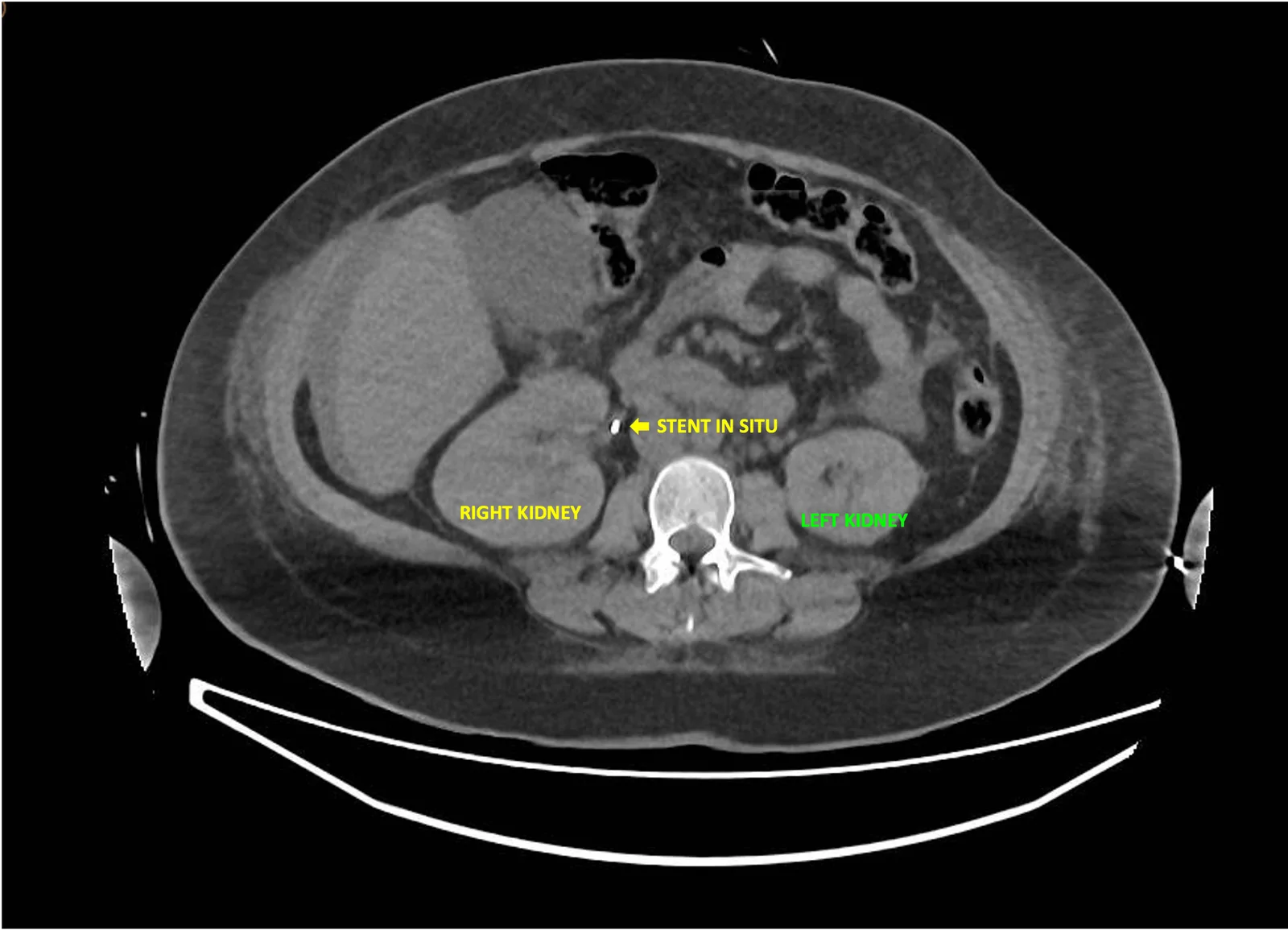
CT scan of the abdomen following right-sided ureteric stenting (yellow arrows showing stent in place), decompression of the right-sided hydronephrosis also evident.

**Table 1:** Serial biochemical trends from admission to recovery and on discharge. CRP: C-reactive protein; eGFR: estimated glomerular filtration rate; NR: not recorded; WCC: white cell count

Hospital day	WCC (ref: 4.0-11.0 × 109/L)	CRP (ref: <10 mg/L)	Serum creatinine (ref: 46-92 μmol/L)	eGFR (ref: ≥90)
Day 1	12.0	11	68	>90
Day 2 (11 hours into admission)	1.8	88	84	71
Day 2 (15 hours into admission)	1.9	NR	123	46
Day 2 (19 hours into admission)	17.8	153	139	40
Day 3	24.8	277	135	41
Day 4	31.0	352	135	41
Day 5	24.9	253	140	39
Day 6	22.9	156	150	36
Day 7	34.7	64	147	21
Day 8	41.6	47	384	12
Day 9	36.0	58	487	9
Day 10	23.3	72	371	13
Day 11	15.9	70	238	21
Day 12	14.4	53	350	14
Day 13	14.7	49	496	9
Day 14 (discharged from the ICU)	16.8	55	589	8
Day 15	12.1	131	618	7
Day 16	8.9	126	539	8
Day 17	9.6	NR	433	11
Day 18	7.4	145	361	13
Day 19	8.0	NR	228	22
Day 21	8.7	41	148	37
Day 23 (discharged from the hospital)	6.7	21	113	51
Day 32 (2-week follow-up)	8.7	<10	79	76
Day 45 (3-week follow-up)	8.3	<10	65	>90

**Figure 5:**
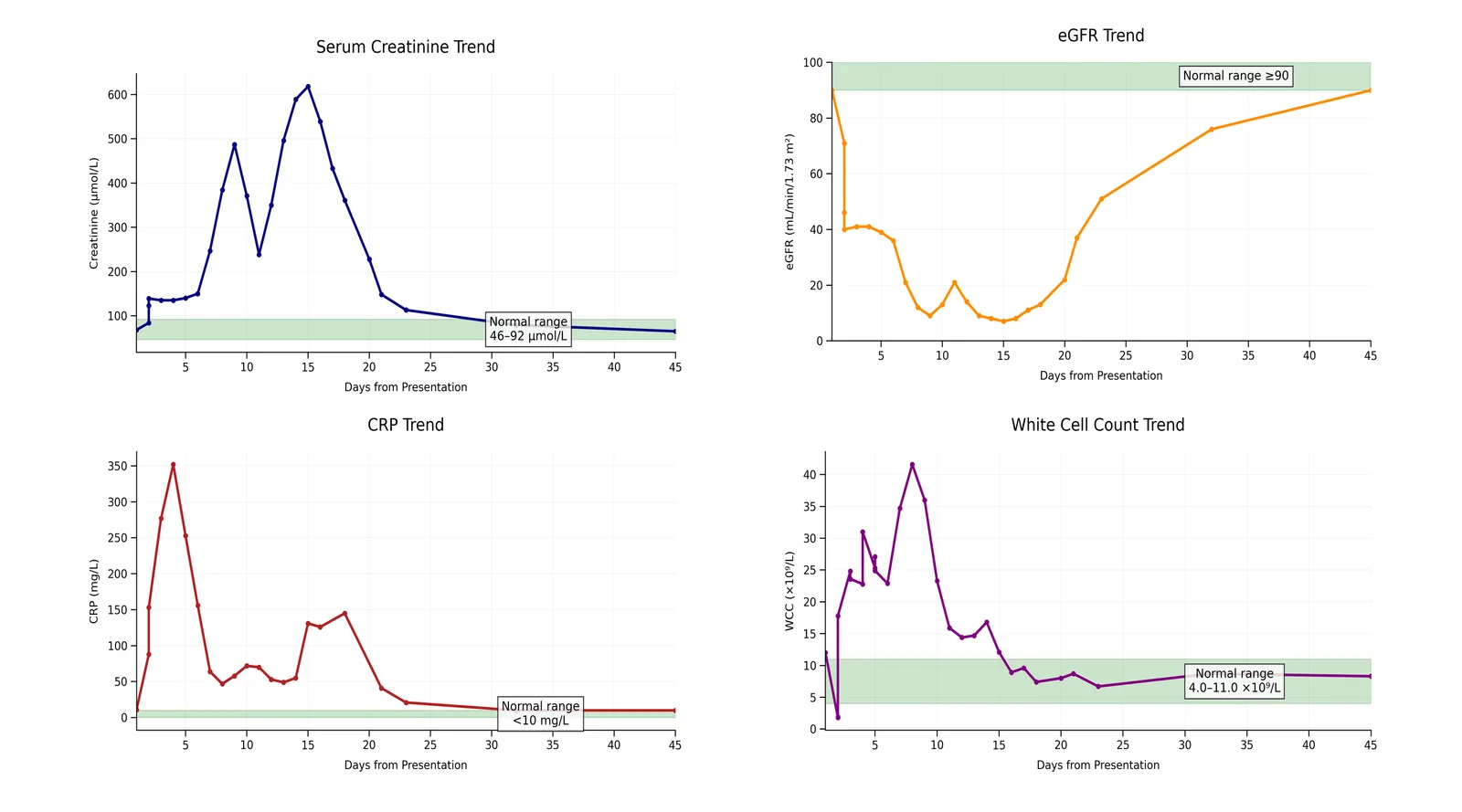
Some significant biochemical markers monitored with trend over the course of admission until full clinical recovery. Normal ranges marked out in green highlight. CRP: C-reactive protein, eGFR: estimated glomerular filtration rate

The patient remained in the ICU for two weeks before being stepped down to the postnatal ward following satisfactory clinical improvement, and her antibiotics were switched to oral co-trimoxazole 480 mg every 12 hours. She was subsequently reviewed by the urology team, who planned definitive stone management with ureteroscopy, laser lithotripsy and ureteric stent exchange following full recovery.

Seven days after discharge from the ICU, both maternal physical recovery and emotional wellbeing had improved sufficiently for discharge home. Ongoing follow-up was arranged with urology, critical care follow-up services and the perinatal mental health team.

At six-month follow-up, the patient reported satisfactory physical and psychological recovery and remained under appropriate specialist follow-up where required.

## Discussion

This case demonstrates how rapidly urosepsis in pregnancy can progress from an apparently routine presentation to life-threatening septic shock. Severe flank pain, previous nephrolithiasis and subsequent physiological deterioration were important indicators that pathology extended beyond physiological hydronephrosis.

Ultrasound remains the recommended first-line imaging modality in pregnancy because it avoids ionising radiation; however, its sensitivity for ureteric calculi is limited. Visualisation may be hampered by the gravid uterus and bowel gas. Consequently, a negative ultrasound does not exclude significant obstruction. When maternal condition deteriorates, and diagnostic uncertainty persists, further imaging, including magnetic resonance imaging (MRI) or low-dose CT when clinically justified, may be warranted [5,6,8].

A central learning point is that infected obstructive uropathy is fundamentally a source-control problem. International sepsis guidance consistently emphasises early source control as a cornerstone of management. In the presence of an infected obstructed collecting system, antibiotic therapy alone is inadequate. Urgent decompression through ureteric stenting or nephrostomy remains the standard of care [6,7,9,10].

The obstetric decision-making in this case was complex. Although delivery does not directly treat maternal sepsis, severe maternal instability coupled with evolving foetal compromise necessitated emergency caesarean birth. The favourable maternal and neonatal outcomes likely reflect timely multidisciplinary decision-making involving obstetrics, anaesthesia, urology, intensive care, microbiology and neonatology [6,11].

The prolonged intensive care course reflects the severity of septic shock. The requirement for invasive ventilation, vasopressor support, renal replacement therapy and management of DIC illustrates the profound systemic consequences of delayed source control in obstructed infection. Recovery following decompression further supports the pivotal role of relief of obstruction in conjunction with antimicrobial therapy [7-10].

Clinicians should remain cautious about attributing severe unilateral loin pain solely to physiological hydronephrosis. Escalation, repeat assessment and consideration of alternative imaging strategies are warranted when symptoms are disproportionate, inflammatory markers rise or maternal observations deteriorate.

## Conclusions

Severe urosepsis in pregnancy represents a time-critical emergency. Although physiological hydronephrosis is common, it may mask clinically significant obstructive uropathy. This case demonstrates the importance of vigilance, repeated reassessment, multidisciplinary collaboration and timely urinary tract decompression. In septic shock secondary to infected obstruction, antibiotics alone are insufficient; prompt source control is essential for maternal and foetal survival.
